# *Drosophila* insulin-like peptide 1 (DILP1) is transiently expressed during non-feeding stages and reproductive dormancy

**DOI:** 10.1038/srep26620

**Published:** 2016-05-20

**Authors:** Yiting Liu, Sifang Liao, Jan A. Veenstra, Dick R. Nässel

**Affiliations:** 1 Department of Zoology, Stockholm University, S-10691 Stockholm, Sweden; 2 INCIA UMR 5287 CNRS, Université de Bordeaux, 33405 Talence Cedex, France.

## Abstract

The insulin/insulin-like growth factor signaling pathway is evolutionarily conserved in animals, and is part of nutrient-sensing mechanisms that control growth, metabolism, reproduction, stress responses, and lifespan. In *Drosophila,* eight insulin-like peptides (DILP1-8) are known, six of which have been investigated in some detail, whereas expression and functions of DILP1 and DILP4 remain enigmatic. Here we demonstrate that *dilp1*/DILP1 is transiently expressed in brain insulin producing cells (IPCs) from early pupa until a few days of adult life. However, in adult female flies where diapause is triggered by low temperature and short days, within a time window 0–10h post-eclosion, the *dilp1*/DILP1 expression remains high for at least 9 weeks. The *dilp1* mRNA level is increased in *dilp2, 3, 5* and *dilp6* mutant flies, indicating feedback regulation. Furthermore, the DILP1 expression in IPCs is regulated by short neuropeptide F, juvenile hormone and presence of larval adipocytes. Male *dilp1* mutant flies display increased lifespan and reduced starvation resistance, whereas in female *dilp1* mutants oviposition is reduced. Thus, DILP1 is expressed in non-feeding stages and in diapausing flies, is under feedback regulation and appears to play sex-specific functional roles.

Insulin and insulin-like growth factor (IGF) signaling (IIS) is part of a nutrient –sensing pathway that regulates multiple aspects of growth, metabolic homeostasis, stress responses, fecundity and lifespan in *Drosophila* and other organisms^1^,^2^,^3^,^4^,^5^,^6^. The IIS pathway is largely conserved over evolution^1^,^7^,^8^,^9^, and therefore *Drosophila* is an excellent genetically tractable model to investigate IIS mechanisms. In *Drosophila* eight insulin-like peptides (DILP1-8) have been identified^1^,^4^,^10^,^11^. Of these, six have been studied in some detail and their spatiotemporal expression patterns and several functions are known. In larvae and adults DILP2, 3 and 5 are produced by a set of 14 median neurosecretory cells in the brain, designated insulin-producing cells, IPCs^1^,^12^,^13^. These DILPs are thought to be released into the open circulation from axon terminations in the corpora cardiaca and corpora allata, as well as on the surface of the anterior intestine and aorta^12^,^13^,^14^,^15^, and have been found sufficient for regulation of stress resistance, fecundity, metabolic homeostasis and lifespan^1^,^2^,^13^,^16^,^17^,^18^. DILP6, an IGF-like peptide, is produced mainly in the fat body and regulates growth during non-feeding stages^19^,^20^. DILP7 is found in a set of about 20 neurons in the abdominal neuromeres of the ventral nerve cord of larvae and adults and plays roles in regulation of gut functions, tracheal growth and reproductive behavior^21^,^22^,^23^,^24^. Finally, DILP8 is released from the imaginal discs of larvae and can delay metamorphosis by inhibiting ecdysone biosynthesis, and thereby coordinates tissue growth with developmental timing^10^,^11^. In adult flies DILP8 and its G-protein-coupled receptor Lgr3 play a role in reproduction^25^. Much less is known about DILP1 and DILP4. According to modENCODE tissue expression transcriptome data^26^ and Slaidina *et al*.^19^ *dilp1* appears to be expressed mainly in midpupal stages and *dilp4* in the embryo. No functions have been revealed for these two DILPs and their cellular location is obscure, although *in situ* hybridization suggested *dilp1* expression in the larval IPCs^13^,^27^. To fill this gap in knowledge we set out to analyze cellular expression and possible functions of DILP1.

Employing novel *dilp1*-Gal4 lines and antisera to DILP1 we found that the expression is in the brain IPCs in the pupa and newly eclosed adult fly. However, in newly-eclosed virgin female flies that had been subjected to low temperature and short photoperiod, and thus entered reproductive diapause^28^, we found that the *dilp1/*DILP1 expression remained high for at least 9 weeks of adult life. This expression declined after one week of recovery from diapause, and DILP1 expression could not be affected by exposure to diapause conditions or low temperature later in life. The DILP1 expression correlated to some extent with the persistence of larval/pupal fat body, and it can be regulated by other DILPs, the neuropeptide short neuropeptide F (sNPF), and inhibited by juvenile hormone. We found a male-specific effect of loss of *dilp1* in normal lifespan and starvation resistance and in females oviposition was reduced in *dilp1* mutants. Thus, the hitherto enigmatic DILP1 seems to play roles distinct from other DILPs and these appear to be partly sex-specific.

## Results

### Cellular expression pattern of *dilp1* and DILP1

**Figure 1:**
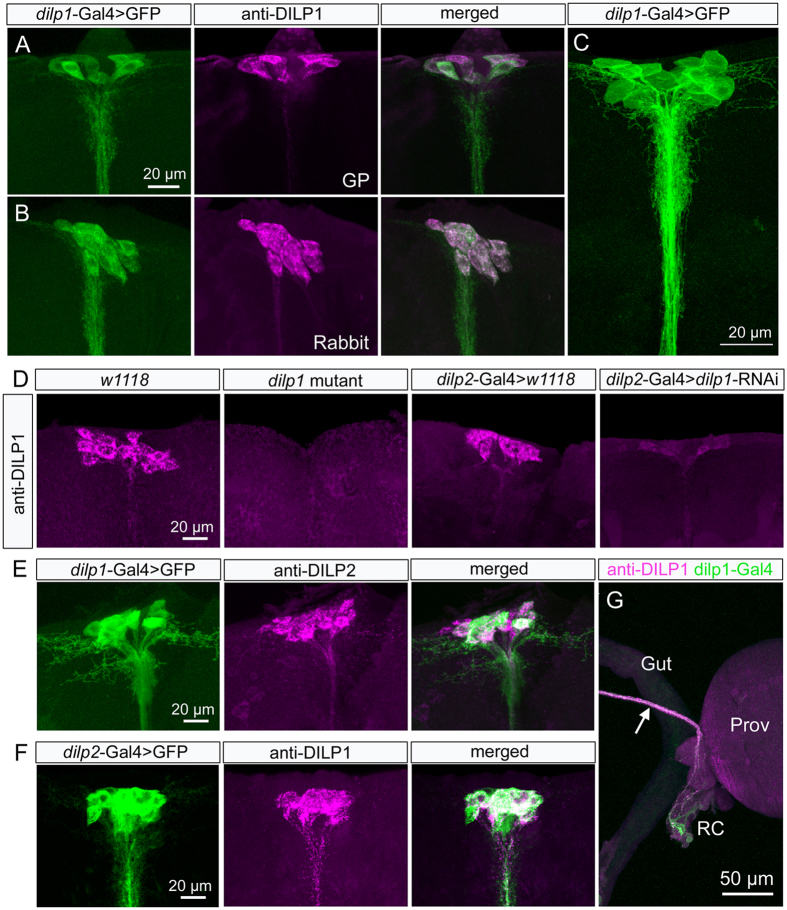
DILP1 is expressed in IPCs in pars intercerebralis of the dorsal brain of newly-eclosed flies. (**A,B**) Antisera to DILP1 produced in guinea pig (GP; **A**) and rabbit (**B**) label neurons identified by *dilp1*-Gal4-driven GFP. Although labeling intensity appears somewhat variable when the two markers are used simultaneously, it is clear that all IPCs coexpress the markers. (**C**) Higher resolution image of the 14 IPCs expressing *dilp1*-GFP. (**D**) DILP1 immunolabeling is not detected in the brain of *dilp1*-mutant flies. Also after *dilp2*-Gal4-driven *dilp1*-RNAi the DILP1 immunolabeling is strongly reduced. (**E**) To confirm that DILP1/dilp1 is co-expressed in DILP2 producing IPCs we applied anti-DILP2 to brains with *dilp1*-Gal4-driven GFP and reveal colocalization of markers. (**F**) Also the reverse experiment with *dilp2*-Gal4 >GFP and anti-DILP1 showed coexpression. (**G**) Details of axon terminations of neurons coexpressing DILP1-immunolabeling and *dilp1*-Gal4-GFP expression in the foregut structures proventriculus (Prov) and retrocerebral complex (RC; corpora cardiaca and corpora allata).

The *Dilp1* precursor gene is unique among the eight *Dilp* genes with its lack of introns^1^,^4^,^29^. Otherwise the amino acid sequence of the precursor displays similarities to those of DILP2, 3 and 5 of the IPCs, and compared to DILP1 precursors of other *Drosophila* species display well-conserved regions corresponding to predicted A- and B-chains and a variable putative C-peptide sequence^4^ (Fig. S1). To obtain DILP1-specific antisera we, thus, selected a sequence of the *Drosophila melanogaster* C-peptide (Fig. S1) coupled to thyroglobulin for immunization. We also generated several *dilp1*-Gal4 drivers (see Methods), two of which are used here. As shown in Fig. 1A–C, the labeling with DILP1 antisera and *dilp1*-Gal4-driven GFP can be seen in the same set of neurosecretory cells in the pars intercerebralis of the brain of newly-eclosed flies. As specificity tests for the antisera we demonstrated that there is no immunolabeling in *dilp1* mutant flies (Fig. 1D) and reduced immunolabeling after *dilp1*-RNAi using *dilp2*- and *dilp5*-Gal4 drivers (Figs 1D and S2A,B). We also observed that pre-immune sera and DILP1 antisera preabsorbed with antigen produced no immunolabeling in the brain. By applying antiserum to DILP1 on brains expressing *dilp2*-driven GFP (Fig. 1E,F), or applying anti-DILP2 to *dilp1*-driven GFP we could determine that DILP1/*dilp1* is expressed in the bona fide IPCs, known to express DILP2, 3 and 5^1^,^13^. The DILP1/*dilp1* expression in IPCs is similar in in newly-eclosed males and females (see Fig. S2D,E).

**Figure 2:**
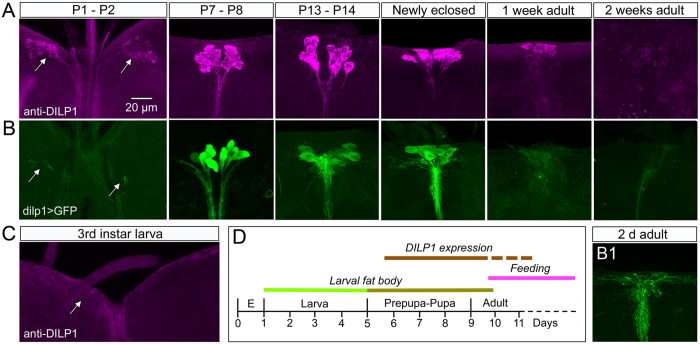
Temporal expression profile of DILP1 immunolabeling and *dilp1*-Gal4 expression in the brain. (**A**) In *Canton S* flies DILP1 immunolabeling appears first in early pupal stages (P1-P2), remains strong in newly eclosed flies and starts fading in 1-week-old flies. (**B**) *Dilp1*-Gal4-driven GFP follows the same expression pattern until flies are newly eclosed. **B1** At about 2–3 days of adult life GFP intensity starts fading. (**C**) In the larval brain no DILP1 or *dilp1* expression could be detected. (**D**) Time course of DILP1 expression in relation to *Drosophila* development, presence of larval fat body and onset of feeding. We did not investigate DILP1 expression during embryonic (E) development.

Next we screened tissues from different developmental stages and tissues (intestine, renal tubules, fat body and gonads) of *Drosophila* (*Canton S*) from first instar larvae to aged adult flies and found that only the brain IPCs express DILP1 immunolabeling or *dilp1*-GFP and that this expression is restricted to certain stages. In wild type flies kept under normal conditions, the DILP1 antiserum labels only the 14 IPCs with an onset in the early to mid-pupa (Fig. 2A). The DILP1 immunoreactivity started to decline after a few days of adult life and could not be detected in any neurons of 2-week-old flies (Fig. 2A). Also the *dilp1*-Gal4-driven GFP is detectable in IPCs from early to mid-pupae and fades to below detection after about one week of adult life (Fig. 2B). Thus, there is a close match between *Dilp1*-GFP expression and DILP1 immunolabeling both spatially and temporarily.

DILP1 seems primarily produced in the IPCs and thus might be coreleased with DILP2, 3 and 5 in the anterior aorta and the retrocerebral complex located in the junction between the intestine and the crop duct (see Fig. 1G). Weaker DILP1 immunolabeling was also detected in branches of the IPCs within the brain dorsally in pars intercerebralis and more ventrally in the tritocerebrum.

The developmental expression of *dilp1*/DILP1 in the IPCs differs from that of DILP2, 3 and 5 immunolabeling, while the latter three can be detected in larvae, throughout pupal development and in both young and old adult flies (Figs S2C and S3). This time course of expression is also seen for transcripts of *dilp2, 3 and 5*^19^. It can be noted that DILP3 and 5 are expressed also outside the brain IPCs in adult flies: DILP5 in ovaries and renal tubules, and DILP3 in midgut muscle cells^16^,^30^,^31^. However, the expression of the novel *dilp5*-Gal4 driver used here is restricted to the IPCs in larvae and adults (Fig. S2), and display a weak expression in muscle fibers of the anterior midgut of adults (not shown).

Our findings suggest that *dilp1*/DILP1 expression is regulated by factors different from those regulating the other peptides of the IPCs and, hence, that DILP1 peptide may serve unique functions. We therefore undertook an investigation of possible factors regulating DILP1 and functional roles of this peptide.

### *Dilp1*/DILP1 expression is maintained high during adult diapause

Newly-eclosed virgin females of *D. melanogaster* can be experimentally triggered to enter reproductive diapause by low temperature provided that the day length is less than 16 h (16L:8D)^32^. Thus, diapause is usually experimentally induced at 11 °C with a slightly shorter photoperiod (10L:14D) and before 10 h post-eclosion^28^,^33^. This dormancy halts ovary development, decreases feeding, alters metabolism, increases stress resistance and drastically extends lifespan^28^. Here we monitored *dilp1* and DILP1 expression in female flies (*Canton S* strain) kept in diapause conditions and sampled over a range of time points (up to 13 weeks), as well as after one week of recovery from three weeks of diapause. The recovery is obtained by transferring diapausing flies to 25 °C and 12L:12D^28^. We monitored relative *dilp1* mRNA expression levels by using quantitative real time PCR (qPCR), and determined DILP1 expression by immunolabeling, as well as GFP fluorescence in *dilp1*-Gal4 >UAS-GFP flies.

**Figure 3:**
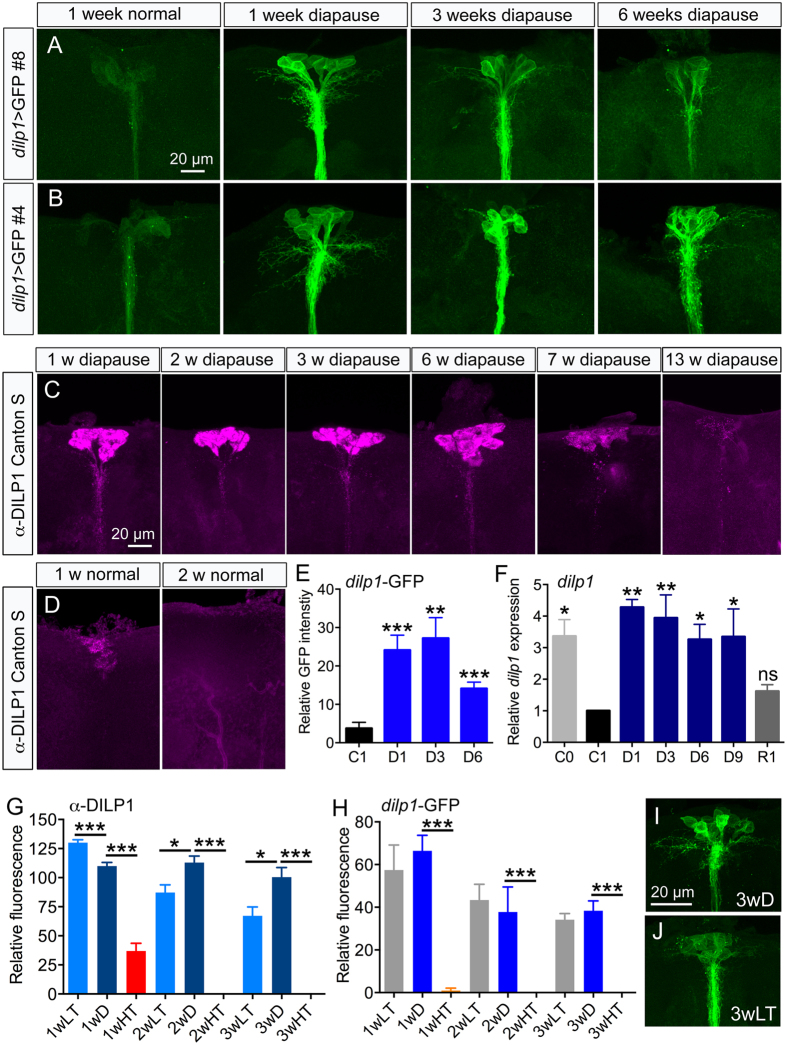
DILP1/*dilp1* remains expressed during adult reproductive diapause. (**A,B**) Two *dilp1*-Gal4 lines #8 (**A**) and #4 (**B**) were used for analysis of GFP expression during diapause. In contrast to 1w old flies kept at 25 °C and 12L:12D, GFP expression remains high over six weeks of diapause. (**C**) In *Canton S* flies DILP1 immunoreactivity could be clearly detected until 7 weeks of diapause. (**D**) Under normal conditions no DILP1 immunolabeling was detected after 2 weeks. (**E**) Relative *dilp1*-GFP fluorescence in cell bodies of IPCs in 1 week controls (C1) and flies diapausing for 1, 3 and 6 weeks (D1–D6). Data are presented as means ± S.E.M, n = 7–15 flies for each time point from three crosses (**p < 0.01, ***p < 0.001, compared to C1, unpaired Students’ *t*-test). (**F**) Relative *Dilp1* transcript levels monitored by qPCR display a similar temporal profile. Newly-eclosed flies (C0) display high expression of *dilp1*, a decrease is seen in one-week-old flies kept in normal conditions (C1), whereas it remained high over 9 weeks of diapause (D1–D9). Flies that had recovered for 1 week (R1) after 3 weeks of diapause displayed control *dilp1* levels (C1). Significance values are shown for comparisons with C1. Data are presented as means ± S.E.M; n = 3 replicates with 10–15 flies in each (*p < 0.05, **p < 0.01, ns – not significant, one way ANOVA followed by Dunnett’s multiple comparisons test). (**G**) DILP1 immunofluorescence levels in *Canton S* flies exposed to 11 °C and 12L:12D (LT) compared to 11 °C and 10L:14D (diapause conditions; D) and 25 °C and 10L:14D (HT) over 3 weeks. Both LT and D treatment induces high DILP1 levels, but not HT. Data are presented as means ± S.E.M, n = 7–12 flies from three replicates (**p < 0.01, ***p < 0.001, compared to D, unpaired Students’ *t*-test). (**H**) Using the same conditions and monitoring *dilp1*-GFP levels yield similar results. Data are presented as means ± S.E.M, n = 6–7 flies from three crosses (**p < 0.01, ***p < 0.001, compared to D, unpaired Students’ *t*-test). (**I**,**J**) Representative images of *dilp1*-GFP expression in flies kept three weeks in diapause conditions (3wD) and low temperature only (3wLT).

We used two different *dilp1*-Gal4 lines to drive UAS-*mCD8*-GFP and recorded GFP fluorescence levels in IPCs after diapause induction. Both fly lines displayed strong fluorescence after 1 and 3 weeks of diapause (Fig. 3A,B), and a slightly reduced, but still high level of fluorescence after 6 weeks of diapause (Fig. 3A,B). The *dilp1*-driven GFP in cell bodies of the IPCs is quantified in Fig. 3E. The Gal4 line #4 displayed the strongest fluorescence and is used in all subsequent experiments. Next we showed that the intensity of DILP1 immunofluorescence decreases in flies after 1 week in non-diapause conditions compared to that of newly eclosed flies (Fig. 3D). However, consistent with *dilp1*-Gal4 driven GFP fluorescence (Fig. 3A,B,E), flies subjected to diapause conditions maintained a strong intensity of DILP1 immunofluorescence over 6 weeks of diapause, and displayed a slight decrease only after 7 weeks and very weak, but detectable, expression after 13 weeks of diapause (Fig. 3C). These experiments show that *dilp1*/DILP1 expression is maintained in IPCs for at least 9 weeks of diapause conditions, whereas in control flies it is lost after about one week of adult life.

We employed qPCR to quantify the *dilp1* transcript levels in diapausing flies at different time points (Fig. 3F). After one week of diapause (D1 in Fig. 3F) flies displayed a 4-fold increase of *dilp1* transcript level compared to one-week-old flies kept in control conditions. Note that recently hatched flies (C0 in Fig. 3F) also express higher levels of *dilp1*, as was shown earlier for *dilp2, 3* and *5*^28^. The dilp1 levels remained high over the 9 weeks of diapause used for measurements. Flies that have been kept for 3 weeks in diapause and then placed in non-diapause conditions for 1-week were shown earlier to recover from diapause as determined by ovarian maturation and several other assays^28^. We monitored *dilp1* transcript in flies that had recovered for one week after three weeks of diapause (R1 in Fig. 3F) and found that the level was back to that seen in one-week-old control flies (C1 in Fig. 3). Previous studies showed that also *dilp2, 3, 5* and *6* were upregulated over 3 or 9 weeks of diapause^28^,^34^ and decreased to initial levels after one week or non-diapause conditions^28^.

**Figure 4:**
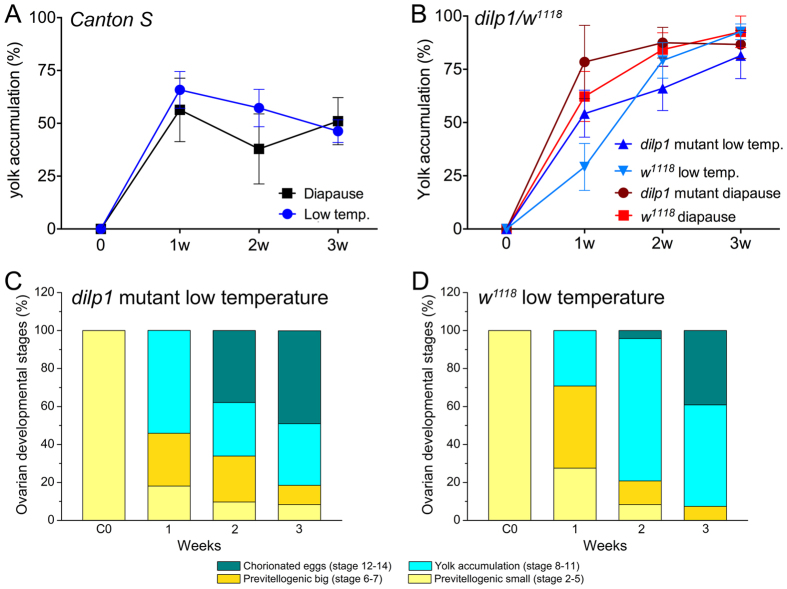
Diapause incidence is not affected in *dilp1* mutant flies. (**A**) Exposure to 11 °C with 12L:12D (Low temp) and diapause conditions induce similar effects on yolk accumulation in Canton S flies over three weeks and suggest that after 3wD about 50% of the flies have entered diapause. (**B**) In *dilp1* mutant flies exposed to the same conditions the effect on yolk accumulation is less strong over 3 weeks than *Canton S* and does not differ significantly from control flies (w^1118^). Data are shown as means ± S.E.M, n = 7–14 flies from 4 independent replicates (ns – not significant, as assessed by two way ANOVA with Bonferroni’s multiple comparisons tests). Note that w^1118^ flies are much less prone to diapause than Canton S^28^. (**C**,**D**) Graphical representation of ovarian development showing defined stages of egg differentiation.

Next we tested exposure of virgin flies to 11 °C with a 12L:12D light regime and 25 °C with 10L:14D and found that 11 °C with the slightly longer day length also induces diapause, as monitored by egg development (see Fig. 4), and leads to prolonged high expression of DILP1 (Fig. 3G,H). However, the short photoperiod had no effect on diapause or DILP1 expression when flies were kept at 25 °C (Fig. 3G–J; see also Figs S4 and S5). To test whether the increased DILP1 expression was due to a general response to low temperature we exposed three week old virgin flies to diapause conditions, to 11 °C alone (with 12L:12D), or to 25 °C with 10L:14D). We found that none of the treatments affected DILP1 levels in 3-week-old flies, i. e. no DILP1 immunolabeling was detected any of the flies (not shown). This is probably because treatments were given outside the diapause-induction time window, from eclosion up to 10 h posteclosion.

Since *dilp1*/DILP1 expression is increased and kept high during diapause, we asked whether genetic knockdown of *dilp1* expression has an effect on diapause induction or maintenance. Thus, we examined diapause incidence in *dilp1* mutant flies by monitoring the rate of vitellogenesis, which is commonly employed to phenotype reproductive diapause in *Drosophila*^28^,^33^,^35^. We compared yolk accumulation in ovaries of *dilp1* mutant flies (in *w*^*1118*^ background) in recently hatched flies and over three weeks of diapause conditions compared to control flies (*w*^*1118*^). There was no significant difference in diapause incidence between mutants and controls when they were exposed to either diapause conditions or 11 °C with 12L:12D (Fig. 4A–D).

### Interactions between DILP1 and other DILPs

**Figure 5:**
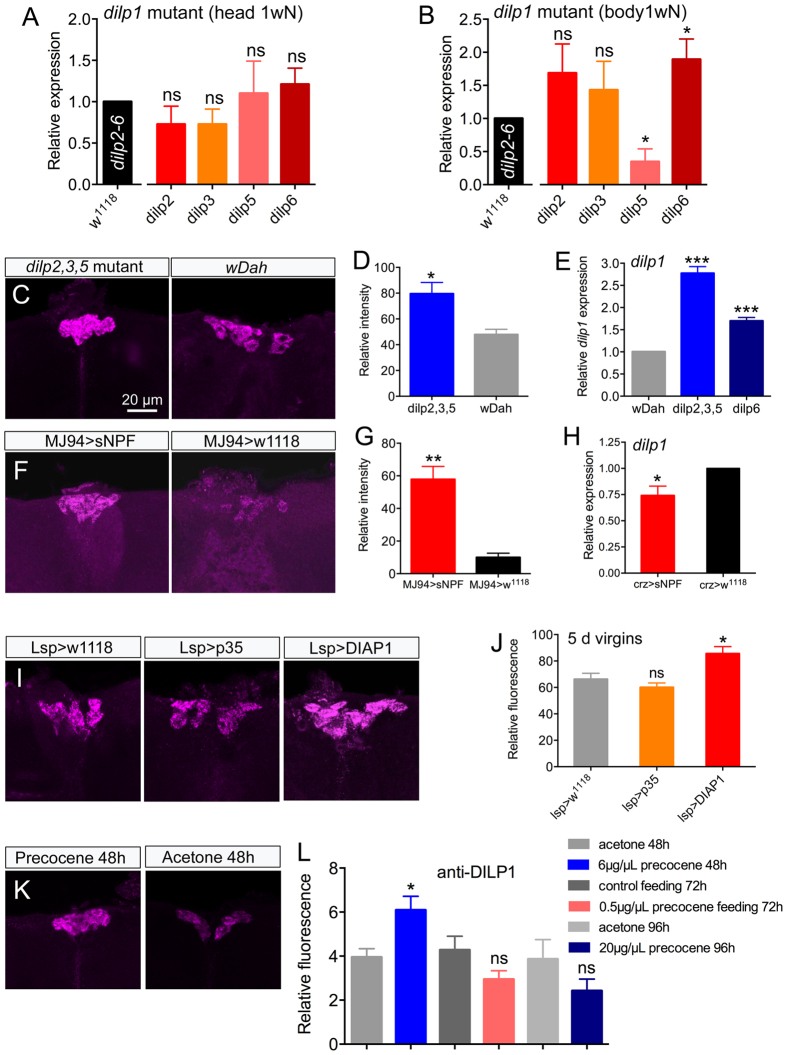
Regulation of *dilp1* levels and effects of *dilp1* mutation on transcription of other *dilps*. (**A,B**) qPCR analysis of one-week-old *dilp1* mutant flies (kept at normal conditions) shows no effects on *dilp2, 3, 5* or *6* transcripts in head extracts (**A**) whereas in whole body (without heads) *dilp5* is downregulated and *dilp6* upregulated (**B**). *dilp* levels are compared to control *w*^*1118*^ flies (the black bar shows control levels of *dilp2, 3, 5* and *6*; set to 1.0); presented as means ± S.E.M, three replicates with 15–20 heads/bodies per replicate (*p < 0.05, unpaired Students’ *t*-test). (**C,D**). In *dilp2, 3, 5* mutant flies DILP1 immunofluorescence is upregulated compared to their controls *w*^*Dahomey*^ (wDah), presented as means ± S.E.M, n = 6–7 flies for each genotype from three crosses (*p < 0.05, unpaired Students’ *t*-test). (**E**) Also *dilp1* is increased in head extracts of *dilp2, 3, 5* and *dilp6*^41^ mutant flies compared to controls (wDah); presented as means ± S.E.M, n = 3 replicates with 15–20 heads in each replicates for each genotype (***p < 0.001, unpaired Students’ *t*-test). (**F,G**) Ectopic expression of sNPF in sensory neurons (MJ94-Gal4 >UAS-sNPF) leads to increased DILP1 immunostaining in IPCs of 1-week old flies; presented as means ± S.E.M, n = 6–7 flies for each genotype from three crosses (**p < 0.01, unpaired Students’ *t*-test). (**H**) Over-expression of sNPF in corazonin-expressing neurons using a *Crz*-Gal4 decreases *dilp1* transcript, but not DILP1 peptide levels (see Fig. S4C,D). (**I,J**) After blocking apoptosis in larval adipocytes by crossing *Lsp*-Gal4 flies to UAS-p35 of UAS-DIAP1 we extended survival of these adipocytes in the adult flies to determine effect on DILP1 immunolabeling in 5d virgin flies. Only Lsp >DIAP1 significantly increased DILP1 fluorescence in adult IPCs; presented as means ± S.E.M., n = 9–10 flies for each genotype from three crosses (*p < 0.05, unpaired Students’ *t*-test). (**K,L**) Precocene 1 applied (in acetone) on abdominal cuticle or fed to the flies halted ovary development. Only when applied topically for 48 h at 6 μg/μL it increased DILP1 immunofluorescence levels; presented as means ± S.E.M, n = 8–13 flies for each treatment from three replicates (*p < 0.05, as unpaired Students’ *t* test).

Expression of some of the DILPs can be regulated by feedback from other DILPs, and it has also been shown that loss of certain DILPs is functionally compensated by others^4^,^36^. Therefore, we tested the effects of loss of *dilp1* on other *dilp* transcripts and also effects of loss of *dilp2, 3, 5* and *dilp6* on *dilp1* expression. Extracts of heads of one-week-old *dilp1* mutant flies did not show any alteration of *dilp2, 3, 5* or *6* transcripts (Fig. 5A), whereas body extracts displayed increased *dilp6* and reduced *dilp5* expression (Fig. 5B). These results suggest that loss of *dilp1* affects *dilp6* in thoracic/abdominal fat body and *dilp5* in ovaries or renal tubules, but has no significant effect on *dilp* transcription in IPCs. However, at the peptide level we found that DILP2 immunolabeling increased slightly in IPCs of *dilp1* mutant flies (Fig. S6A,B), possibly suggesting decreased release of this peptide. As a comparison we monitored effects of loss of *dilp1* on *dilp* transcripts in late pupae (stage P14) when *dilp1* is normally high. At this stage *dilp1* mutants displayed a slight decrease in *dilp3* and *dilp6* and no change in *dilp2* and *5* (Fig. S2F).

Next we analyzed *dilp1* expression in one-week-old flies after loss of *dilp2, 3, 5* in triple mutants. Indeed, the loss of the three other DILPs of the IPCs triggered a slight, but significant, increase in DILP1 immunofluorescence in IPCs (Fig. 5C,D), and a strong increase of *dilp1* transcript (Fig. 5E). We also analyzed *dilp1* transcript in *dilp6* mutant flies, since an earlier study indicated that *dilp1* and *dilp6* display compensatory regulation^19^. The *dilp1* transcript increased significantly in *dilp6* mutants compared to control flies (Fig. 5E).

### Regulation of DILP1 by short neuropeptide F (sNPF)

A few neuropeptides and neurotransmitters have been shown to act on the IPCs to regulate production and/or release of DILPs^14^,^37^,^38^. One of these, short neuropeptide F (sNPF), is also known to regulate feeding in *Drosophila*^27^,^39^. An earlier study has reported that mis-expression of short neuropeptide F (sNPF) in specific sensory neurons induces upregulation of *dilp1* and *dilp2* in the larval CNS^27^. Here we targeted expression of sNPF to the same set of sensory neurons using the MJ94-Gal4 and monitored DILP1 immunofluorescence in IPCs of one-week-old adult flies. The sNPF expression led to a significant increase of DILP1 immunofluorescence compared to control flies (Fig. 5F,G). Some of the sensory neurons of MJ94-Gal4 send their axonal projections to the pars intercerebralis close to the presumed dendrites of the IPCs (Fig. S6E), but it is not clear whether MJ94 expressing neurons act directly on IPCs. Nevertheless, sNPF from these neurons seems to regulate DILP1 production also in early stages of adult life.

Another study has shown that neurons in the pars lateralis, designated DLPs, express sNPF and impinge on the brain IPCs^40^. That study demonstrated that targeted knockdown of sNPF in the DLPs of adult male flies lead to diminished expression of *dilp2* and *dilp5* mRNA. Thus, we tested whether overexpression of sNPF in these neurons had an effect on *dilp1* levels in female flies. We found that there was a very small, but significant, decrease in *dilp1* transcript (Fig. 5H). However increased sNPF expression in DLPs did not result in a significant change in DILP1 immunolabeling (Fig. S6C,D), suggesting that DILP1 production, but not release, was affected.

### Timing of *dilp1*/DILP1 expression with regulation of organismal development and differentiation and ovary maturation

Interestingly, the timing of the *dilp1*/DILP1 expression not only correlates with non-feeding stages, but also coincides with organismal remodeling during pupal development, adult eclosion, as well as physiological and reproductive maturation starting during the first day of adult life. The extensive tissue reorganization and growth during pupal development requires a reallocation of resources from nutrient stores in adipocytes and histolysing larval tissues; this requires activation of IIS and growth factor signaling^19^,^20^,^41^. Furthermore, during this period several hormonal signals are required to orchestrate tissue growth and differentiation^42^,^43^ and we thus sought evidence for links between DILP1 and other hormonally regulated events, including resource reallocation.

During the first day of adult life food intake is very low and steady state metabolite and hormone levels have not yet reached those seen in mature flies^28^. Another characteristic of the pupa and the first day of adult life is the presence of fat body cells derived from the larva^44^,^45^. These dissociated adipocytes constitute an energy and nutrient store during pupal development and also serve the newly eclosed adult until it is ready for flight, food search and feeding, which requires wing expansion and tanning of cuticle^44^,^45^. When no longer needed, the larval adipocytes undergo apoptosis the first days of adult life^44^. To test whether the presence of larval fat body is correlated with *dilp1*/DILP1 expression we delayed apoptosis in larval adipocytes by expressing anti-apoptosis genes (p35 and DIAP1) using an *Lsp*-Gal4 driver specific for fat body. When measuring DILP1 fluorescence in IPCs of 5 day old flies we found that expression in *Lsp* >DIAP1 flies was significantly higher than in controls and in *Lsp* >p35 flies (Fig. 5I,J). Thus, a slight effect of delaying adipocyte death can be seen on DILP1 expression. The apoptosis of larval adipocytes is ecdysone dependent^44^ and we investigated a possible link between DILP1 and ecdysone signaling by determining levels of prothoracicotropic hormone (PTTH) in brain lateral neurosecretory cells in *dilp1* mutant flies. At adult eclosion PTTH triggers ecdysone production in the prothoracic glands and a few days post-eclosion the PTTH producing neurosecretory cells are no longer detectable^46^,^47^. In newly eclosed (1–3 hours after adult eclosion) *dilp1* mutant flies the PTTH immunolabeling is significantly lower than in controls, suggesting increased PTTH release (Fig. S7).

We next tested whether ovarian maturation, which also occurs the first days of adult life, correlates with *dilp1*/DILP1 expression. Ovarian maturation and vitellogenesis depends on active juvenile hormone (JH) signaling. Exposing newly eclosed virgin flies to precocene 1, an inhibitor of JH biosynthesis in corpora allata, leads to inhibition of vitellogenesis and oocyte growth in ovaries^48^,^49^. We applied precocene 1 in acetone (6 μg/μL or 20 μg/μL) to the abdomen of newly eclosed virgin flies (*Canton S*) and monitored DILP1 immunofluorescence in 48 h and 96 h old flies compared to controls exposed to only acetone. Ovary development was arrested for both concentrations (not shown), but only the lower concentration led to increased DILP1 immunolevel (Fig. 5K,L). Feeding precocene to the flies (0.5 μg/μL in normal food) also resulted in blocked ovary development in 72 h old flies, but had no effect on DILP1 expression (Fig. 5K,L). Thus, there seems to be a dose-dependent effect of blocking JH signaling, but the relation of DILP1 to ovarian development is not clear. We also tried to prevent diapause (induce vitellogenesis) and diminish DILP1 expression by feeding or topical application of the JH analog Methoprene^50^. Thus, we tested (1) female flies (*Canton S*) that had been kept for two weeks in diapause with food containing 1.5 mM Methoprene, and (2) flies that after three weeks of diapause had 0.5 μL of 1 mM Methoprene in acetone applied to the abdomen and put back in diapause conditions for one week. As shown in Fig. S8 neither treatment altered the DILP1 expression in the IPCs.

**Figure 6:**
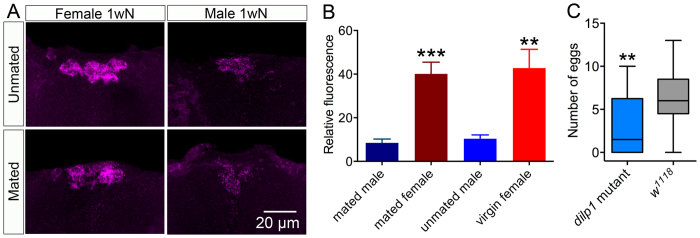
Roles of DILP1 in reproduction and fecundity. (**A**) There is a sex dimorphism in strength of DILP1 immunolabeling in IPCs of one-week-old mated and unmated flies. (**B**) Quantification of DILP1 immunolabeling in one-week-old mated and unmated flies, showing the significantly higher DILP1 expression in female IPCs in both mated and unmated flies. Data are presented as means ± S.E.M, n = 7–9 flies for each genotype from three independent replicates (***p < 0.001, **p < 0.01, ns – not significant, as assessed by unpaired Students’ *t*-test). (**C**) Box-Whisker graph showing that the number of eggs laid is significantly lower in *dilp1* mutant than in control flies. Data are presented as medians ± range (where boxes show 25–75% percentile and the population median), n = 21–30 flies from three independent replicates (**p < 0.01, as assessed by Mann-Whitney test).

We asked whether mating had an effect on DILP1 expression in male and female flies. Thus, one-week-old mated flies were monitored, but no significant difference was detected in DILP1 labeling of IPCs in mated and unmated specimens (Fig. 6A). However in both states female flies had significantly stronger DILP1 expression in IPCs than males (Fig. 6A,B). This was not the case in newly-eclosed flies (Fig. S2D). To search for a role of DILP1 in ovary development we monitored the rate of oviposition in *dilp1* mutant and control flies. The *dilp1* mutant flies displayed a significantly reduced oviposition (Fig. 6C).

### Effect of *dilp1* mutation on lifespan and stress resistance

**Figure 7:**
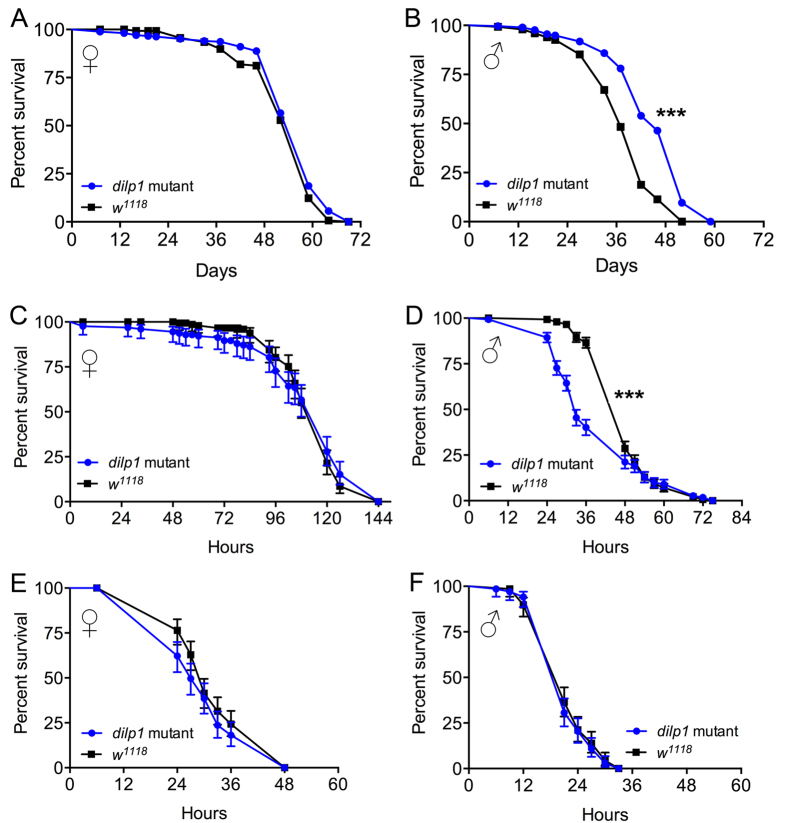
Male *dilp1* mutant flies display altered lifespan and stress resistance. (**A,B**) Lifespan is significantly increased in mated male *dilp1* mutant flies compared to controls (w^1118^), whereas mated mutant females display no phenotype. Data are presented in survival curves, n = 291 and 149 flies for *dilp1* mutant and w^1118^ (***p < 0.001, as assessed by Log-rank (Mantel-Cox) test). (**C,D**) Survival is drastically decreased only in mated male *dilp1* mutant flies exposed to starvation. Data are presented in survival curve, n = 134 and 149 flies for *dilp1* mutant and w^1118^ (***p < 0.0001, as assessed by Log-rank (Mantel-Cox) test). (**E,F**) Neither female nor male *dilp1* mutants react to desiccation (dry starvation) by altered lifespan. Data are presented in survival curve, n = 128 and 141 flies for female *dilp1* mutant and w^1118^ as well as n = 137 flies each for male *dilp1* mutant and w^1118^ (***p < 0.0001, as assessed by Log-rank (Mantel-Cox) test).

Diminished insulin signaling extends lifespan in *Drosophila*, and ablation of the brain IPCs or knockdown of *dilp2* is sufficient to increase longevity^2^,^4^,^51^,^52^. We monitored longevity in male and female *dilp1* mutant flies under normal feeding conditions and registered an increase of median lifespan in males only (Fig. 7A,B).

Flies with ablated IPCs display an increased resistance to starvation^2^. To investigate possible DILP1 function in stress resistance, we tested responses to starvation and desiccation in *dilp1* mutant flies. In these tests 4–5d old *Dilp1* mutant flies were tested with *w*^*1118*^ flies as controls.

Before testing effect of starvation on survival, we monitored DILP1 immunolevels in one-day-old female flies (*Canton S*) that were starved for 24 h and found a significant decrease in immunolabeling (Fig. S6G), maybe suggesting increased DILP1 release. Next we showed that only male *dilp1* mutant flies display reduced resistance to starvation, as seen in decreased survival, (Fig. 7C,D) and the response to desiccation was not affected in either sex (Fig. 7E,F). Thus, there may be a sexual dimorphism in DILP1 function in relation to lifespan and stress tolerance.

## Discussion

Until now the spatiotemporal expression and function of DILP1 in *Drosophila* was largely unknown. Our study shows that under normal rearing conditions both *dilp1* transcript and DILP1 peptide are expressed transiently by a set of 14 brain IPCs during pupal stages and the first days of adult life. However, a continuous high *dilp1*/DILP1 expression can be seen in IPCs of female flies that have entered reproductive diapause. The DILP1 expression, thus, coincides with the non-feeding stages of pupae and the very young fly^44^,^45^, and the strongly reduced feeding during adult diapause^28^. This temporal expression of DILP1 suggests a specific function of the peptide that may be related to development, growth or energy homeostasis characteristic of states of minimal nutrient intake.

The arrest of feeding at the end of the larval life is programmed and continues throughout pupal development. Thus, cell growth and proliferation during this stage occurs without global growth or gain of mass, using stored nutrients and supplies derived from histolysis of obsolete larval tissues^19^,^41^. The fat-body-derived DILP6 is known to promote tissue growth during non-feeding stages and *dilp6* is induced by ecdysone in the wandering third instar larva^19^,^20^. Possibly DILP1 is functionally accessory to DILP6 during this non-feeding period, although a very minor growth promoting role was detected^4^. An additional possibility is a link to stages with no reproductive activity. Characteristic of *Drosophila* pupae, newly eclosed flies, and diapausing adults, is the immature ovaries and lack of vitellogenesis (see^28^). Thus, DILP1 down-regulation could correlate with both the metabolic transition during the first days of adult life and the onset of sexual maturation. Indeed, we found that *dilp1* mutant flies display a reduced early oviposition, suggesting a role in egg development. This is consistent with the importance of intact systemic insulin signaling in the control of ovarian germ line stem cells^53^.

To further understand DILP1 function we undertook an analysis of factors regulating its expression and effects of manipulations of DILP1 production. It has been shown that loss of certain DILPs can be compensated by upregulation of others^4^,^19^,^36^. We tested the effect of loss of *dilp2, 3, 5* and *dilp6* using mutant flies^4^ and found that the *dilp1* transcript was elevated in both mutants. Conversely, we detected a slight but significant increase in *dilp6* expression and a reduced *dilp5* expression in the body, but not heads, of 1-week-old *dilp1* mutant flies. These findings suggest that loss of *dilp1* may affect *dilp5* transcription in ovaries or renal tubules and *dilp6* in adipocytes of the body. Apparently the loss of *dilp1* did not affect *dilp2* and *dilp3* levels (or *dilp5* in the head), suggesting that in the IPCs there are no detectable compensatory transcriptional mechanisms for lack of *dilp1* in adult flies. The upregulation of *dilp1*/DILP1 after loss of *dilp2, 3, 5* or *dilp6* may be reminiscent of the situation during diapause where the *dilp1* transcript is elevated but systemic insulin signaling appears to be generally downregulated, probably due to diminished DILP release (see^28^,^34^,^54^).

Previous studies have shown that a few neuropeptides and neurotransmitters act on the IPCs to alter their activity and/or production of DILPs (summarized in^14^,^37^,^55^,^56^). One of the peptides acting on IPCs is sNPF, released from DLP neurons, that triggers increased levels of *dilp2* and *dilp5* transcripts in the adult brain^40^. In another study sNPF was ectopically targeted to sensory neurons of larvae using the MJ94-Gal4 and it was shown that *dilp1* and *dilp2* transcripts were elevated^27^. When we targeted sNPF to MJ94 neurons there was an increase in DILP1 immunolabeling of the IPCs of one-week-old flies, but we noted no effect on peptide levels when targeting DLP neurons. However, measuring *dilp1* transcript we noted a slight decrease after sNPF overexpression in DLP neurons. These findings suggest that sNPF signaling is involved in regulation of DILP1/*dilp1* expression. Earlier work has suggested multiple functional roles of sNPF, including regulation of food intake, modulation of food odor responses, fine control of locomotor behavior, and learning and memory^57^,^58^,^59^,^60^,^61^,^62^.

There might, hence, be a link between the role of sNPF in feeding and food odor processing and its action on DILP1 production and/or release in the IPCs. This regulation can perhaps be extended to taste inputs since flies lacking gustatory bristles due to a null mutation in the gene *Pox neuro* (*Poxn*) were shown to live longer and in females display increased levels of *dilp1, dilp3* and *dilp6*^63^. The MJ94-Gal4 line used in our study includes gustatory receptors^64^,^65^ and may, thus, account for the effect of sNPF overexpression on *dilp1* levels. The study of *Poxn* mutants revealed a female-specific upregulation of *dilp1, dilp3* and *dilp6* and a male downregulation of *dilp3* and *dilp5*, suggesting a sex-specific influence of taste on lifespan mediated by different DILPs^63^.

An intriguing finding in our study is that flies kept in reproductive diapause display a sustained high *dilp1*/DILP1 expression in IPCs until the dormancy is interrupted. Adult diapause is characterized by halted reproduction, a drastic reduction of food intake, accompanied by a shift in energy metabolism towards increased nutrient stores, as well as increased stress tolerance, and upregulated immune defense^28^,^34^,^54^. In *Drosophila* diapause can only be triggered in newly eclosed flies before 10 h of adult life^28^,^32^. Flies at this stage share several features with the late pupae in addition to the lack of food intake and high expression of DILP1/*dilp1*^28^,^44^,^45^. For instance, both pupae and adult flies that are less than 24 h old display presence of fat body cells derived from the larva^44^,^45^. These dissociated adipocytes constitute an energy and nutrient store during pupal development and also serve the newly eclosed adult until it is ready for flight, food search and feeding, which requires wing expansion and tanning of cuticle^44^,^45^. The presence of larval fat body cells is extended somewhat in diapausing flies. Thus, we tested whether genetic blocking of apoptosis in larval fat body in non-diapausing flies has an effect on DILP1 expression. Similar to the study of Aguila *et al*.^44^ we could only extend the presence of larval adipocytes a few days, but found that DILP1 expression was increased in 5d old flies compared to controls.

The sustained DILP1 expression during diapause is not likely to be due only to the presence of larval fat body since this is no longer detectable after 3 weeks of diapause (unpublished observations). Since ovaries do not mature during diapause we tested whether some ovary-derived factor might be involved in the regulation of *dilp1*/DILP1 expression. By treating newly eclosed flies with precocene 1, an inhibitor of juvenile hormone biosynthesis, we halted ovary maturation and observed a dose-dependent increase in DILP1 expression in young flies. However, since we did not obtain clear evidence for long term effects of larval fat body or ovary maturation in keeping the DILP1 expression high, we suggest that during diapause the DILP1 level in IPCs remains high due to the altered metabolic state and endocrine signaling (including diminished general IIS) characteristic of dormancy^28^. It can be noted that loss of *dilp1* does not result in drastically altered diapause incidence, probably due to redundant functions of other DILPs. However, *dilp1* mutant flies display reduced egg-laying, indicating a role of the peptide in ovary development, maybe accessory to DILP2 which was shown to control germ line stem cells in *Drosophila* ovaries^53^.

In our work we found that *dilp1* mutant male flies display increased median lifespan under normal conditions, and also reduced resistance to starvation. This is similar to the effects of deletion of the IPCs, which renders flies more long-lived^2^, but the reduced starvation resistance in *dilp1* mutants is opposite of findings for IPC ablation and *dilp6* mutants^2^,^19^. Our experiments herein do not provide any clues to this sex dimorphism in the role of *dilp1*. It is noteworthy that deletion of single *dilps*, except *dilp2*, has little effect on fly physiology^4^. Thus, *dilp1* mutants display unexpectedly strong effects on adult male physiology, in spite of the dilp1/DILP1 expression peaking during pupal stages. One possible explanation is that loss of *dilp1* seems to have very limited effects on compensatory expression of *dilp2, 3* and *5* in IPCs and has rather small effects on *dilp5* and *6* transcripts in the body. In other words, compensatory interactions between *dilp1* and other *dilps* work primarily one-way whereby *dilp1* levels are affected by knockout of *dilp2, 3, 5* and *dilp6.* This is different for *dilp2* mutants, which display increased *dilp3* and *dilp5* transcript^4^,^36^. Another aspect of interest is that *dilp1*/DILP1 expression is very weak in flies older than one week (kept at 25 °C) and yet we see effects on lifespan and stress responses in older male *dilp1* mutant flies. Therefore it is possible that the adult phenotypes are consequences of developmental effects induced by lack of DILP1 signaling in the pupa and/or first 1–2 days of adult life, similar to findings for DILP6^19^.

In summary, our study is the first to determine the spatial-temporal expression pattern of DILP1. The expression appears to be primarily in the brain IPCs where DILP1 is colocalized with DILP2, 3, 5 and the CCK-like peptide drosulfakinin (DSK)^1^,^13^,^66^. In contrast to DILP2, 3, 5, 6 and 7, we find that under normal conditions DILP1 is produced by only in the pupa and first few days of adult life. However during adult reproductive diapause DILP1 levels remain high. These findings indicate that DILP1 has a unique function that may be relevant in stages where food intake is low or absent and where sexual maturity has not been reached. Analysis of *dilp1* mutant flies reveals that the peptide is important in formation of adult male responses to starvation and general longevity, and in female ovary maturation.

## Methods

### Fly lines and husbandry

As wild type and control flies we used *Drosophila melanogaster* of the strains *Canton S* and *w*^*1118*^, respectively, from The Bloomington *Drosophila* Stock Center (BDSC), Bloomington, IN. Three mutant strains were employed: *dilp1* (transcript null allele), *dilp6^41^* (small deletion covering first exon; loss of functional allele) and *dilp2, 3, 5* mutants^4^, kindly provided by S. Grönke (Cologne, Germany). As controls for *dilp1* mutant, we used *w*^*1118*^ and for *dilp6*^41^ and *dilp2, 3, 5* mutants controls were *w*^*Dahomy*^, dependent on mutant background, all from S. Grönke. We utilized MJ94-Gal4 (from L. Griffith, Waltham, MA), UAS-*dilp1*-RNAi from The Vienna *Drosophila* Resource Center, Vienna (VDRC), UAS-2xsNPF (from K. Yu, Daejon, Korea), *dilp2*-Gal4 (from E. Rulifson, Stanford, CA), *Crz*-Gal4 (from J.H. Park, Knoxville, TN), *Lsp*-Gal4 (BDSC), *Ppl*-Gal4 (from M.J. Pankratz, Bonn, Germany), UAS-mCD8-GFP, UAS-DIAP1 and UAS-p35 (from BDSC). The production of two sets of *dilp*-Gal4 lines is described in the next section.

Parental flies for Gal4-UAS crossings were reared on BDSC food medium with 1.5 g/L nipagin (http://flystocks.bio.indiana.edu/Fly_Work/media-recipes/bloomfood.htm). *Canton S* flies and progeny of Gal4-UAS crossings were (if not otherwise indicated) raised on food medium containing 100 g/L sucrose (S), 50 g/L yeast (Y), 12 g/L agar, 3 mL/L propionic acid and 3 g/L nipagin (2S:1Y food medium). Flies were kept either under normal conditions with 12:12 Light:Dark cycle at 25 °C or under diapause conditions with 10:14 Light:Dark cycle at 11 °C. In the starvation and desiccation experiments on *dilp1* mutants food medium containing 2S:1Y was used for raising flies.

### Production of *dilp1-*Gal4 and *dilp5-*Gal4 lines

In both the *dilp1* and *dilp5* genes the transcription start site is very close the predicted TATA box, making it difficult to amplify the putative promoters in a single PCR reaction. We thus used two sequential PCR reactions. For the first PCR reaction primers for *dilp1* were

dilp1-1(5′-GGACAGAATTCACCGTCAGCGGATAATCGTA-3′) and

dilp1-2 (5′-TGCTGGCTAAACATCTTGGA-3′)

and for dilp5

dilp5-1 (5′-GGACAGAATTCGGCTCTGTTCGGGATTGATA-3′) and

dilp5-2 (5′-ATCATTGCCTTGCTGGAACT-3′).

The products of these PCR reactions were then reamplified by primers dilp1-1 and dilp1–3 (5′-GGATCGGATCCTTGGATATGCAGTGAATGCTC-3′) and dilp5-1 and dilp5-3 (5′-GGATCGGATCCTTGCTGGAACTGCTGCTG-3′) respectively. After gel purification and digestion with Eco RI and Bam HI the putative promoters were cloned into the P[pAKH-GAL4] vector^67^, from which the AKH promoter had been removed with the same enzymes. Sequencing confirmed that the transgenes had the intended sequences. Embryos were injected by thebestgene.com yielding 10 independent transformants for *dilp1* and 10 for *dilp5*.

Two transformants (#4 and #8) for *dilp1* were selected after screening GFP expression patterns driven by *dilp1*-Gal4 throughout larval, pupal, newly eclosed and 1-week-old fly stages. Similarly, two transformants for *dilp5* were selected for further use here (only few experiments).

### Production of DILP1 antisera

For generation of antisera we selected a sequence from the C-peptide of *D. melanogaster* DILP1 synthesized with a cysteine in the N-terminus: CEVQDDSSMWQTLDGAGYS. This peptide was conjugated to maleimid-coupled thyroglobulin via the SH group of the N-terminal cysteine and the conjugate used for immunization of two rabbits and two guinea pigs. The production of the antigen and the immunization were performed by Pineda Antibody Service (Berlin, Germany). Preimmune sera were collected from each animal prior to immunization.

### Other antisera and immunocytochemistry

We used rabbit antisera to A-chains of DILP2 and DILP3^30^ and C-peptide of DILP5^31^ at a dilution of 1:2000. A rabbit antiserum to *Drosophila* prothoracicotropic hormone (PTTH^68^), was kindly provided by Dr. P. Leopold (Nice, France) and applied at 1:500. The central nervous system of third instar larvae, different pupal stages and adult CNS, as well as various tissues (fat body, gut, ovaries) were fixed in ice-cold 4% paraformaldehyde (4% PFA) in 0.1 M sodium phosphate buffer (PB; pH 7.4) for 2–4 h. After washing with PB 3 × 15 min, tissues were dissected in PB. All tissues were washed finally in 0.01 M phosphate buffered saline (PBS) with 0.25% Triton-X (PBS-Tx) for 15 min. Tissues were then incubated in primary antibodies for 24–48 h at 4 °C with gentle agitation. After washes (4 × 15 min) in PBS-Tx, secondary antibodies were applied for overnight or 48 h at 4 °C. Tissues were then washed in PBS-Tx 7 × 10 min, rinsed in 0.01 M PBS and mounted with 80% glycerol in 0.01 M PBS. Rabbit and mouse anti-GFP (1:1000) were used (Invitrogen, Carlsbad, CA). The following secondary antibodies (1:1000) were employed for detection: goat anti-rabbit Alexa 546, goat anti-rabbit Alexa 488, goat anti-mouse Alexa 488, goat anti-mouse Alexa 546 (all from Invitrogen), Cy3-tagged goat anti-guinea pig antiserum (Jackson ImmunoResearch, West Grove, PA).

### Quantitative real-time PCR (qPCR)

Total RNA was extracted from whole flies or separated heads and bodies of experimental virgin female flies using Trizol-chloroform (Sigma-Aldrich) from three independent biological replicates with 15–25 flies in each replicate. Quality and concentration of the RNA were determined with a NanoDrop 2000 spectrophotometer (Thermo Scientific). For cDNA synthesis reactions 2 μg of total RNA, 0.4 μL random hexamer primer (Thermo Scientific) and 2 μl of M-MuLV reversible transcriptase (Thermo Scientific) were used. The cDNA was then applied for quantitative real-time PCR (qPCR) using a StepOnePlus™ System (Applied Biosystem, USA) instrument and SensiFAST SYBR Hi-ROX Kit (Bioline) according to the manufacturer. For each sample triplicate reactions of the total volume of 20 μl were conducted with a primer concentration of 400 nM and 4 μl of diluted 1:10 cDNA template. The mRNA levels were normalized to rp49 levels in the same samples. Relative expression values were determined by the 2^−ΔΔCt^ method^69^.

We used the following primers for qPCR (all displayed 5′–3′):

*dilp1* F: CGGAAACCACAAACTCTGCG

*dilp1* R :CCCAGCAAGCTTTCACGTTT

*dilp2* F: AGCAAGCCTTTGTCCTTCATCTC

*dilp2* R: ACACCATACTCAGCACCTCGTTG;

*dilp3* F: TGTGTGTATGGCTTCAACGCAATG

*dilp3* R: CACTCAACAGTCTTTCCAGCAGGG;

*dilp5* F: GAGGCACCTTGGGCCTATTC

*dilp5* R: CATGTGGTGAGATTCGG

*dilp6* F: CCCTTGGCGATGTATTTCCCAACA

*dilp6* R: CCGACTTGCAGCACAAATCGGTTA

*rp49* F: ATCGGTTACGGATCGAACAA

*rp49* R: GACAATCTCCTTGCGCTTCT

### Diapause induction and analysis of ovarian development

Diapause was induced by keeping virgin female flies, collected 4–6 h post-eclosion, at 11 °C and a slightly shorter photoperiod (10L:14D)^28^,^33^. At each investigated time point of diapause (as well as in control and recovery groups) ovarian development was monitored microscopically and used as a criterion for degree of reproductive diapause as proposed earlier^33^,^48^,^70^. Ovaries were dissected in 0.1 M phosphate saline buffer (pH 7.4) and their developmental stages assessed by using the combined criteria of several investigations^28^,^33^,^48^,^71^,^72^. Stages of ovarian development were divided into four groups: small previtellogenic ovaries (stage 2–5), big previtellogenic ovaries (stage 6–8), accumulated yolk (stage 9–11) as well as several chorionated eggs (stage 12–14). Recovery from diapause was monitored one week after transferring flies from diapause conditions to 25 °C and 12L:12D; this period is sufficient for onset of vitellogenesis and reversal of hormonal and metabolic status back to pre-diapause values^28^. Results are displayed as percentage (%) of ovaries in different development stages, or as incidence (%) of yolk accumulation in analyzed ovaries. Data are shown as means of 4 independent replicates with 7–14 flies in each replicate.

### Stress assays

For starvation resistance experiments, 4–5d-old flies were placed in vials containing 0.5% aqueous agarose for starvation (no access to food) in an incubator at 25 °C with 12:12 h Light:Dark (LD) conditions and controlled humidity. Each vial contained 15 flies to avoid a crowded environment. Dead flies were counted in every 12 hours. For desiccation resistance the experiment was the same, except that flies obtained no food and no water.

All these experiments were performed in three replicates with at least 30 flies of each genotype per replicate. Survival curves were produced in Prism GraphPad 6.0.

### Precocene 1 and methoprene application and feeding

Precocene 1 (Sigma-Aldrich, catalog no. 195855-1G) was diluted in acetone with final concentrations 6 μg/μL and 20 μg/μL and applied on the abdomens of newly eclosed virgin flies with a small brush (5 μL was applied to each abdomen). After precocene treatment flies were checked for recovery (locomotion, wing expansion and flipping). For 96 h treatment precocene 1 was applied a second time after 48 h. Control flies were exposed to acetone alone. As a second approach newly eclosed virgin flies were fed precocene 1 mixed into food medium (final concentration 0.5 μg/μL). Control flies were fed food medium alone.

Methoprene (Sigma-Aldrich, St Louis, MO, PESTANAL 33375, racemic mixture), a JH analog^50^, was diluted in acetone with final concentrations of 1.5 mM or 1 mM. For topical application 0.5 μL of 1 mM methoprene was quickly applied on the abdomens of virgin flies after three weeks of diapause and flies were transferred back into vials and the diapause incubator as soon as possible to minimize the interruption of diapause. Flies after one week in diapause were taken for immunohistochemistry. For feeding methoprene 2S:1Y food medium supplemented with 1.5 mM methoprene was given to newly eclosed virgin flies kept for two weeks in diapause.

### Oviposition

For oviposition, the number of eggs laid by individual female flies within 16 h was counted. Individual pairs of newly eclosed males and females were mated for 6 days. Egg numbers from 21–30 pairs of flies were counted 16 hours after fly pairs were placed into separate food vials.

### Statistical Analysis

All statistical analyses were performed using Prism GraphPad 5.0. Survival data were analyzed by Log rank test with Mantel-Cox posttest. For the remaining experiments, data was first checked with Shapiro-Wilk normality test and then analyzed with unpaired Students’ *t* test or ANOVA with Dunnett’s or Sidak’s multiple comparisons tests. If data was not normally distributed, non-parametric tests, Mann Whitney or Kruskal-Wallis test was performed.

## Additional Information

**How to cite this article**: Liu, Y. *et al. Drosophila* insulin-like peptide 1 (DILP1) is transiently expressed during non-feeding stages and reproductive dormancy. *Sci. Rep.* **6**, 26620; doi: 10.1038/srep26620 (2016).

## Supplementary Material

Supplementary Information
