# Supplementary material for: Drosophila insulin-like peptide 1 (DILP1) is transiently expressed during non-feeding stages and reproductive dormancy
Source: Sci Rep. 2016 May 20;6:26620. doi: 10.1038/srep26620 (PMC4873736; doi:10.1038/srep26620)
Supplement: Supplementary Information [file srep26620-s1.pdf]

Supporting material files

Drosophila insulin-like peptide 1 (DILP1) is transiently expressed during non-feeding stages and reproductive dormancy

Yiting Liu, Sifang Liao, Jan A. Veenstra, Dick R. Nässel

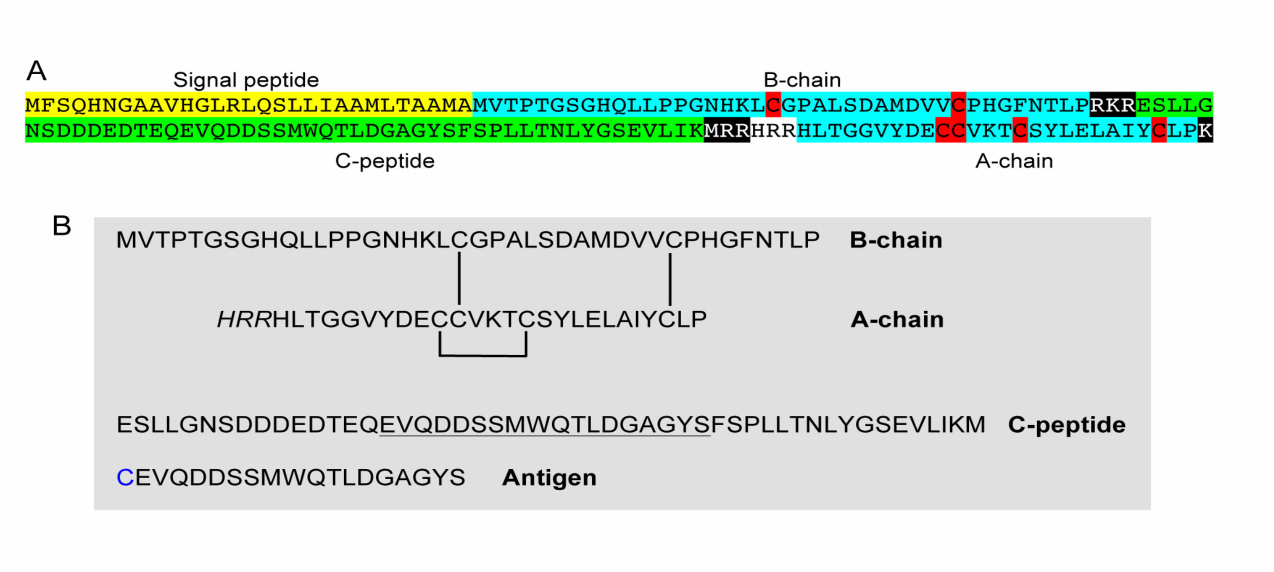

Fig. S1. Amino acid sequence of DILP1 precursor and predicted mature DILP1 peptide. **A.** DILP1 precursor with signal peptide and B and A chains, as well as C-peptide. The cysteines are indicated in red. **B**. Predicted structure of DILP1 with disulphide bridges. The C-peptide and sequence used for antigen (underlined) are shown.

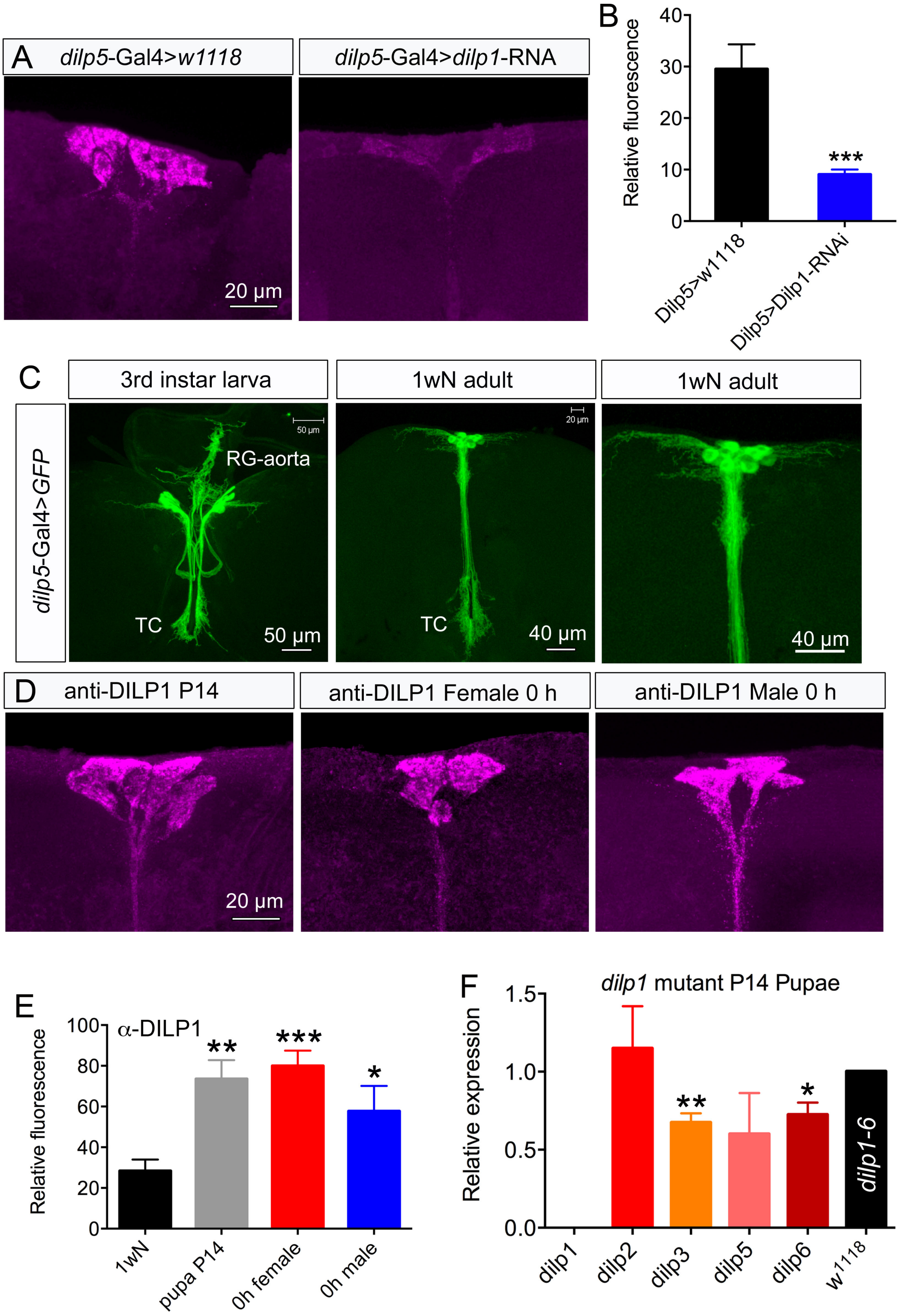

Fig. S2. Expression of DILP1/*dilp1* in pupae and early male and female adults. **A-B.** DILP1 immunolabeling diminishes after *dilp1*-RNAi with a *dilp5*- Gal4 driver compared to control (*dilp5*-Gal4>w1118). Data are presented as means ± S.E.M, n = 6-8 flies for each genotype from three crosses (***p<0.001, as assessed by unpaired Students' t-test). **C**. The *dilp5*-Gal4 drives GFP in IPCs of third instar larva and adults (1 week old flies shown). Note axon terminations in ring gland and aorta (RG-aorta) and branches in tritocerebrum (TC). **D.** DILP1 immunolabeling in late pupa (P14) and newly-eclosed (0 h) female and male flies. **E.** Quantification of DILP1 immunofluorescence in one-week-

old flies (1wN), late pupae (P14) and newly-eclosed flies (0h). Data are presented as means ± S.E.M, n = 6-8 flies from three replicates (*p<0.05, **p<0.01, ***p<0.001, as assessed by unpaired Students' t-test). **F.** Expression of *dilp* mRNAs in *dilp1* mutant pupae (stage P14). The black bar represents control levels (set to 1.0) of dilp1, 2, 3, 5, and 6 in w^{1118} pupae. Only *dilp3* and *dilp6* levels diminished significantly. Data are presented as means ± S.E.M, n = 3 independent replicates with 15- 20 pupae in each replicates for each genotype (*p<0.05, **p<0.01, as assessed by unpaired Students' t-test).

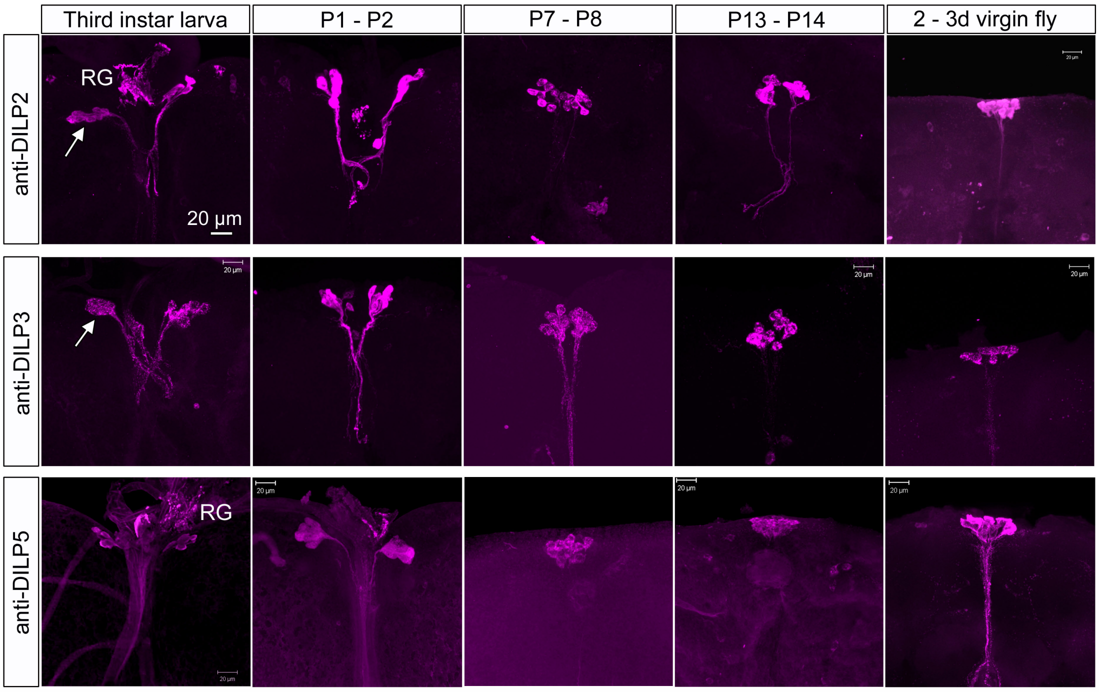

Fig. S3. Expression of DILP2, 3 and 5 immunolabeling during development. DILP2, 3 and 5 immunolabeling can be seen in larvae as well as all stages of the pupa and in 2-3d old adult. In published accounts it is well known that DILP2, 3 and 5 immunolabeling remain high also in older flies.

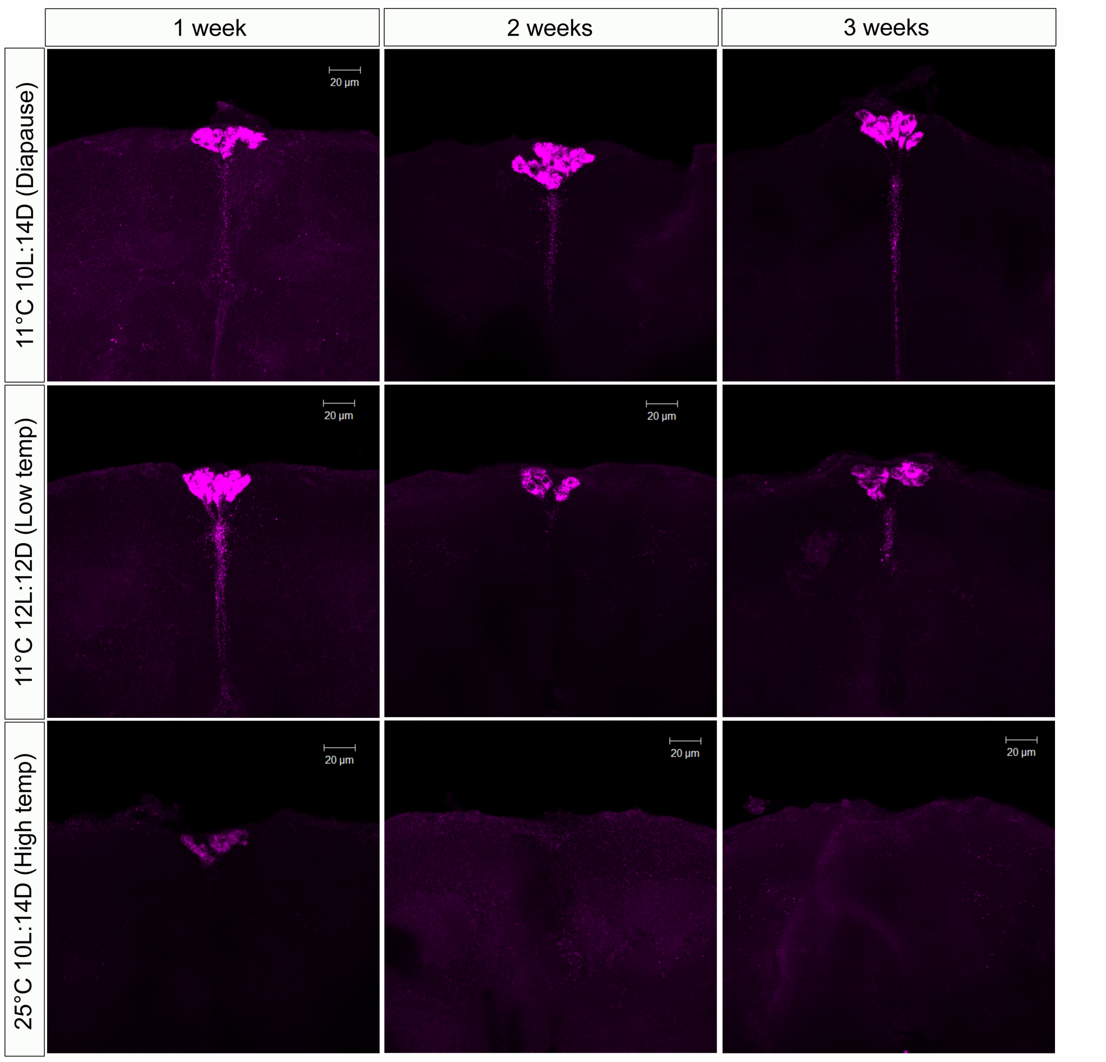

Fig. S4. DILP1 expression in IPCs is affected by low temperature, but not short photoperiod. Comparison of DILP1 immunolabeling in IPCs exposed to 11°C and 10L:14D (diapause conditions), 11°C and 12L:12D (low temp), and 25°C and 10L:14D (high temp) for 1 - 3 weeks. Accompanying quantification graph in Fig. 3G.

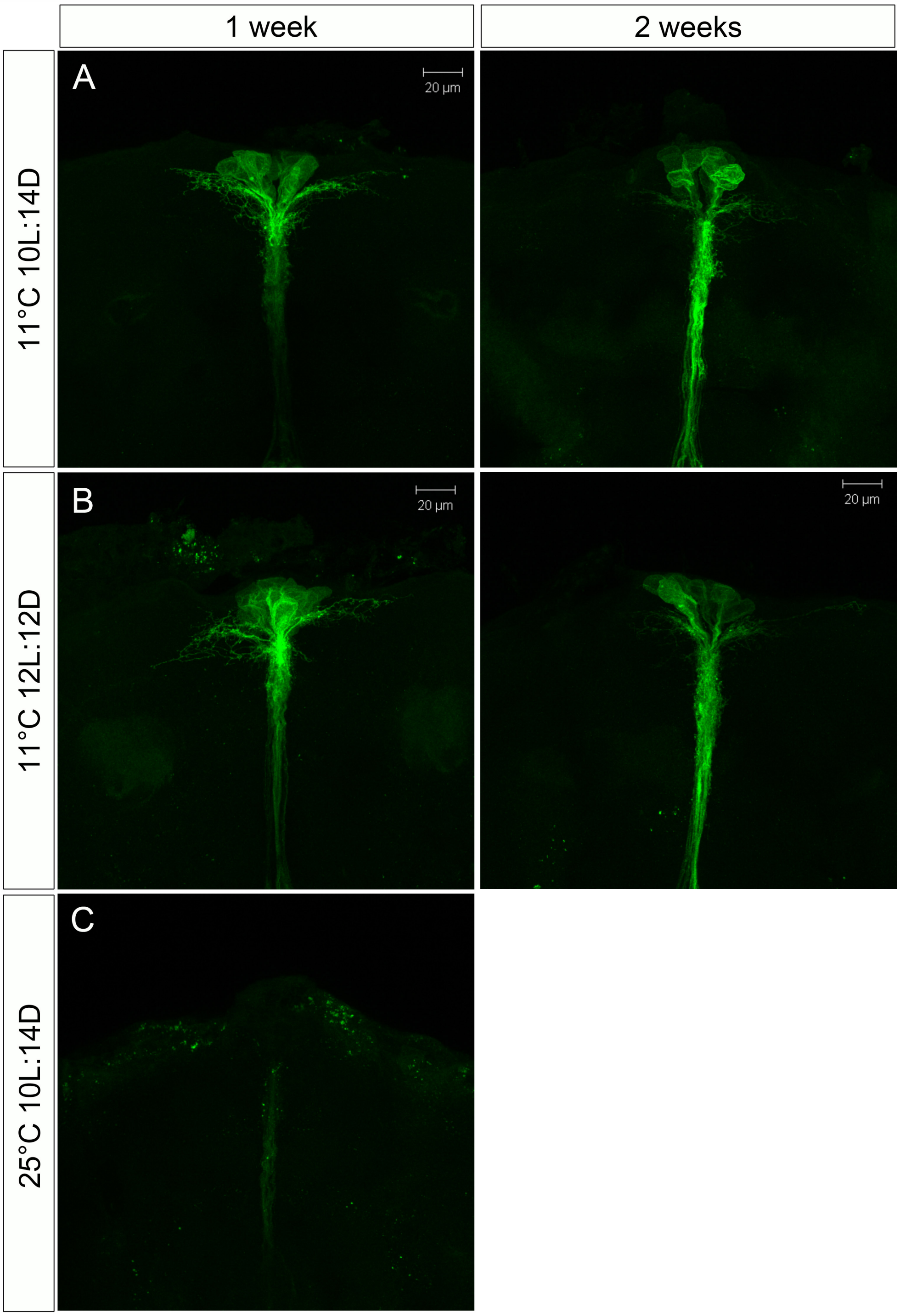

Fig. S5. The *dilp1*-GFP expression in IPCs is affected by low temperature, but not short photoperiod. Comparison of *dilp1*-GFP expression in IPCs exposed to 11°C and 10L:14D (diapause conditions), 11°C and 12L:12D, and 25°C and 10L:14D for 1 - 2 weeks (3 weeks exposure are shown in Fig. 3 I, J). Accompanying quantification graph in Fig. 3H.

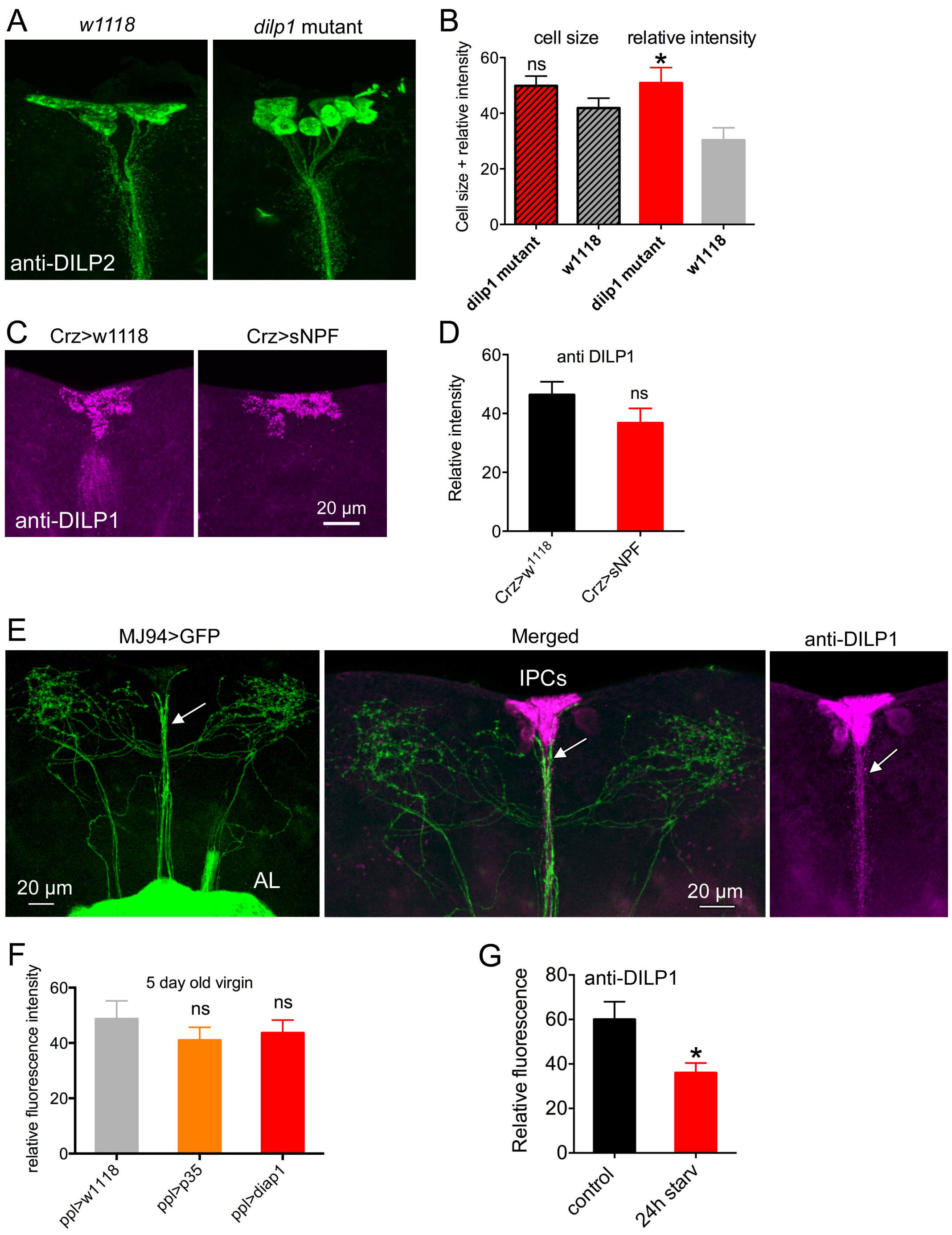

Fig. S6. Manipulations of DILP1 and DILP2 levels and lack of effects. **A-B**. In dilp1 mutant flies the intensity of DILP2 immunolabeling in IPCs is increased, but the size of the IPC cell bodies is not affected. Data are presented as means ± S.E.M, n = 6-8 flies for each genotype from three replicates (*p<0.05, ns - not significant, as assessed by unpaired Students' t test). **C-D.** Targeted over-expression of sNPF in corazonin-producing neurons (Crz>sNPF) has no effect on DILP1 immunolabeling. Data are presented as means ± S.E.M, n = 8-9 flies for each genotype from three crosses (ns – not significant, as assessed by unpaired Students' t-test). **E**. The MJ94-Gal4 expressing neurons are sensory neurons (including olfactory and gustatory neurons), some of which impinge on the DILP1 immunoreactive IPCs along the brain mid-line (arrow). The antennal lobe (AL) is massively labeled by MJ94-GFP. **F.** Using the pumpless (ppl) Gal4 driver to express p35 and diap1 has no effect

on DILP1 immunolevels in 5-day-old virgin flies (in contrast to the Lsp1-Gal4 driver shown in Fig. 5I-J). Data are presented as means ± S.E.M, n = 10-11 flies for each genotype from three crosses (ns - not significant, as assessed by unpaired Students' t-test). **G**. DILP1 fluorescence is decreased after 24 h starvation in newly-eclosed virgin Canton S flies. Data are presented as means ± S.E.M, n = 8-10 flies from three replicates (*p<0.05, as assessed by unpaired Students' *t*-test).

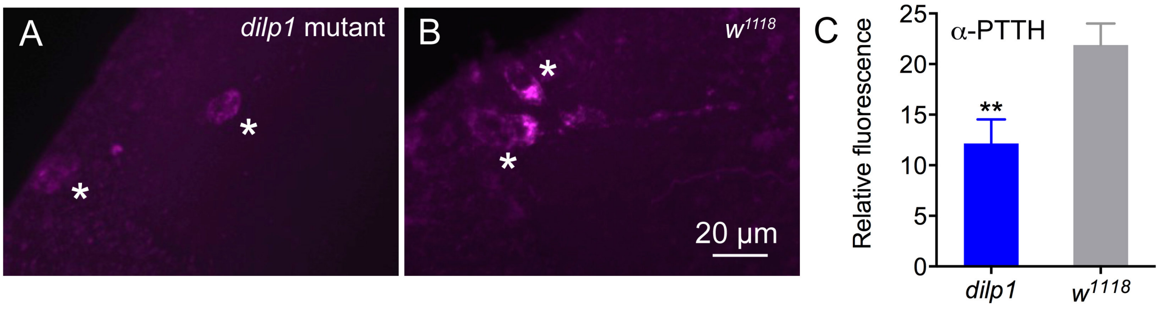

S. Fig. 7. The level of the neuropeptide PTTH decreases in 1 to 3 h old *dilp1* mutant flies. **A-B**. PTTH immunolabeling in lateral neurons of the brain is weaker in *dilp1* mutant flies (**A**), than in w^{1118} controls (**B**). **C.** Quantification of PTTH immunofluorescence. Data are presented as means ± S.E.M, n = 13 for *dilp1* mutant and n= 12 for w^{1118} (**p<0.01, as assessed by unpaired Students' *t*-test).

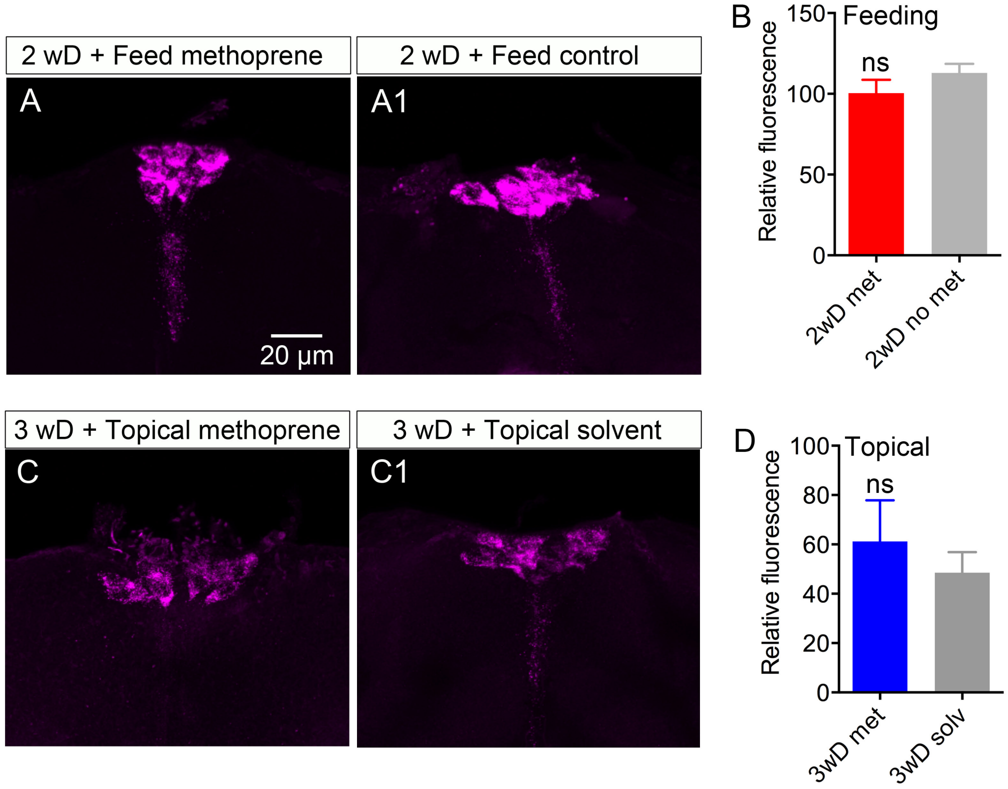

Fig. S8. Methoprene treatment does not affect DILP1 expression in IPCs. **A** and **B.** Feeding the JH analog methoprene for two weeks to diapausing flies (2wD) does not affect DILP1 immunolabeling. **C** and **D**. Topical application of methoprene to abdomens of 3-week diapausing flies (3wD) has no effect. Data are presented as means ± S.E.M, n = 5-7 flies from three replicates (ns – not significant, as assessed by unpaired Students' *t*-test).
